# Co‐creating an Outcome Measure for Social Robots in Dementia Care

**DOI:** 10.1002/alz70858_101127

**Published:** 2025-12-25

**Authors:** Susanna E. Martin, Katelyn A. Teng, Julie M. Robillard

**Affiliations:** ^1^ University of British Columbia, Vancouver, BC, Canada; ^2^ BC Children's and Women's Hospital, Vancouver, BC, Canada; ^3^ BC Children's and Women's Hospital, Vancouver, BC, Canada

## Abstract

**Background:**

Social robots continue to demonstrate their potential to reshape dementia care and improve quality of life for persons living with dementia (PLWD). However, despite their increasing popularity, gaps remain in our understanding of how robots make a difference: stronger evidence is needed. Here we report on the first of a three‐phase project to co‐create an evidence‐based, patient‐reported outcome measure (PROM) to capture the impact of social robots in dementia care.

**Methods:**

All aspects of this project are informed by an older adult advisory group called The League (*N* = 8 members). Ten co‐creation workshops with healthcare providers, PLWD, and care partners, were carried out online over Zoom (*n* = 5), and in‐person at long‐term care facilities (*n* = 5). Participants (*n* = 51) shared their perspectives on how social robots could impact the experience of living with dementia. Transcripts from the discussions were analyzed for emerging themes and data from polls were collated to identify the outcomes that ranked most important for including in the PROM tool.

**Results:**

Three key themes emerged as prominent areas of life in which social robots can have an impact: (1) emotional wellness, (2) physical health, and (3) social interactions. Participants also expressed that the PROM should measure outcomes related to a social robot's impact on independent functioning in daily activities and capture potential implications to a user's safety and privacy. Participants shared preferences for a short‐form PROM that is quick to complete, written in plain language and administered online or in a paper format.

**Conclusion:**

Results from the co‐creation workshops are currently used in the development and validation of a PROM that captures the most salient and meaningful outcomes for users of social robots living with experience of dementia. This program of work serves to inform best practices and policies in the growing use of social robotic technology in dementia care as well as underscoring the value of engagement with PWLD as valuable co‐creators in health technology research.

